# Is geographical variation driving the transcriptomic responses to multiple stressors in the kelp *Saccharina latissima*?

**DOI:** 10.1186/s12870-019-2124-0

**Published:** 2019-11-21

**Authors:** Cátia Marina Machado Monteiro, Huiru Li, Kai Bischof, Inka Bartsch, Klaus Ulrich Valentin, Erwan Corre, Jonas Collén, Lars Harms, Gernot Glöckner, Sandra Heinrich

**Affiliations:** 1 Marine Botany, Faculty Biology/Chemistry, University of Bremen, Bremen, Germany; 2 Station Biologique de Roscoff, plateforme ABiMS, CNRS: FR2424, Sorbonne Université (UPMC), 29680 Roscoff, France; 3 Sorbonne Université, CNRS, Integrative Biology of Marine Models (LBI2M), Station Biologique de Roscoff, 29680 Roscoff, France; 4 Fisheries College, Ocean University of China, Qingdao, China; 5 Alfred-Wegener-Institute, Helmholtz Centre for Marine and Polar Research, Bremerhaven, Germany; 6 Institute for Biochemistry I, Medical Faculty, University of Cologne, Cologne, Germany; 7 Institute for Plant Science and Microbiology, University of Hamburg, Hamburg, Germany

**Keywords:** Gene expression, Brown algae, Temperature stress, Salinity stress, Geographical variation, RNA-seq

## Abstract

**Background:**

Kelps (Laminariales, Phaeophyceae) are brown macroalgae of utmost ecological, and increasingly economic, importance on temperate to polar rocky shores. Omics approaches in brown algae are still scarce and knowledge of their acclimation mechanisms to the changing conditions experienced in coastal environments can benefit from the application of RNA-sequencing.

Despite evidence of ecotypic differentiation, transcriptomic responses from distinct geographical locations have, to our knowledge, never been studied in the sugar kelp *Saccharina latissima* so far.

**Results:**

In this study we investigated gene expression responses using RNA-sequencing of *S. latissima* from environments with contrasting temperature and salinity conditions – Roscoff, in temperate eastern Atlantic, and Spitsbergen in the Arctic. Juvenile sporophytes derived from uniparental stock cultures from both locations were pre-cultivated at 8 °C and S_A_ 30. Sporophytes acclimated to 0 °C, 8 °C and 15 °C were exposed to a low salinity treatment (S_A_ 20) for 24 h. Hyposalinity had a greater impact at the transcriptomic level than the temperature alone, and its effects were modulated by temperature. Namely, photosynthesis and pigment synthesis were extensively repressed by low salinity at low temperatures. Although some responses were shared among sporophytes from the different sites, marked differences were revealed by principal component analysis, differential expression and GO enrichment. The interaction between low temperature and low salinity drove the largest changes in gene expression in sporophytes from Roscoff while specimens from Spitsbergen required more metabolic adjustment at higher temperatures. Moreover, genes related to cell wall adjustment were differentially expressed between Spitsbergen and Roscoff control samples.

**Conclusions:**

Our study reveals interactive effects of temperature and salinity on transcriptomic profiles in *S. latissima*. Moreover, our data suggest that under identical culture conditions sporophytes from different locations diverge in their transcriptomic responses. This is probably connected to variations in temperature and salinity in their respective environment of origin. The current transcriptomic results support the plastic response pattern in sugar kelp which is a species with several reported ecotypes. Our data provide the baseline for a better understanding of the underlying processes of physiological plasticity and may help in the future to identify strains adapted to specific environments and its genetic control.

## Background

Kelps are brown macroalgae of the order Laminariales that dominate the rocky benthic ecosystems in temperate to polar regions. They play an important ecological role by contributing greatly to the primary production in coastal systems and by providing shelter and nursery for many animal species [1, 2]. Furthermore, they hold economic value, namely for food, feed, and raw material for pharmaceutical industries and are of growing interest for the aquaculture sector [3–5].

*Saccharina latissima* (L.) C.E. Lane, C. Mayes, Druehl, et G. W. Saunders is a species of Laminariales that has a wide latitudinal range in the Northern hemisphere from polar to temperate waters [6]. In the eastern Atlantic, it is distributed from the Arctic (> 80°N) to the north of Portugal (41.5°N) [7]. This suggests that the species has a high potential to adapt to variations in temperature and irradiance but the level of adaptation along latitudinal gradients is not clear [8, 9]. The optimum growth range for *S. latissima* is between 10 °C and 15 °C; above 20 °C the mortality rate is high [10, 11]. Concomitantly, *S. latissima* exhibits optimum growth at absolute salinities (S_A_) between 23 and 31, shows a strong reduction around S_A_ 13 and a high mortality below S_A_ 8 [12, 13].

The physiological tolerance to several biotic and abiotic stress factors is well-known in *S. latissima* e.g. [1, 8–12, 14]; however, the underlying molecular bases remain poorly understood; respective studies have been limited to temperature, irradiance, and UV radiation [15–17].

The variable environments along its distributional range have created ecotypic differentiation within *S. latissima*, which were documented between populations from Helgoland, Germany and Spitsbergen, Norway [8, 9]; and between populations of the core and southern limit distribution in the western Atlantic along the coastline of the USA [18]. Furthermore, recent studies indicate high genetic differentiation between Spitsbergen and Brittany, France populations and absence of admixture based on microsatellite genotypic data [6, 19]. In this study, we investigate how contrasting environments of origin (Roscoff, France, temperate Atlantic and Spitsbergen, Norway, Arctic) may be correlated with transcriptomic responses in *S. latissima*. Despite inhabiting a typical marine coastal environment in Roscoff, algal exposure to low salinities may occur occasionally at spring low tides in association with rain or runoff from land. In contrast, in Spitsbergen, in the Arctic, exposure to low salinities may be more frequent due to freshwater run-off from ice and glacier melting in the fjords [20]. In a changing climate, increased ice-melting and precipitation events may even amplify frequency and duration of exposure to hyposalinity conditions in the North Atlantic [21]. Moreover, these two locations are characterized currently by very different temperature conditions over the course of the year – *S. latissima* specimens living in Spitsbergen experience temperatures between approx. 0 °C to 8 °C [22] while specimens in Roscoff live in a warmer environment with mean seawater temperatures ranging between 9 °C and to 15 °C [23].

We hypothesized that sporophytic material isolated from these contrasting locations will show considerable differences in gene expression when exposed to the same abiotic stress conditions. We expected that material from Spitsbergen is better adapted to a combination of low temperature and low salinities while Roscoff material would perform better under high temperature and full marine salinity. Moreover we hypothesized that interactions between temperature and salinity might even enhance the differences between the response patterns of sporophytes from both locations.

## Results

Read mapping, transcriptome quality assessment and Principal Component Analysis.

The number of reads per library ranged from 28 to 38 million with an average of 33 million reads. From the three transcriptome assemblies produced, the transcriptome assembly based on Roscoff samples was selected given that it presented the best overall remapping rates (~ 85%) compared to the cDNA library assembly based on Spitsbergen samples (~ 83%) and hybrid assembly created from both libraries (~ 83%). More precisely 85.2% of reads of the Roscoff cDNA library and 85.8% of reads of the Spitsbergen cDNA library remapped to the “Roscoff” de novo transcriptome. Additional mapping tests against the closest available genome of *Saccharina japonica* [24] led to a much lower remapping rate (39%) than on the de novo assembly. The raw transcriptome assembly consisted of 205,363 transcripts (or 135,959 Trinity genes) with an average contig size of 760 bp. Despite the apparent verbosity of the transcriptome, 90 % of total expression was present in 28,001 of transcripts (14% of the full transcriptome). The results of the contamination search revealed a very low percentage of potential contamination in our transcriptome (bacteria – 0.4%, oomycetes – 0.4%); therefore we did not remove any sequences. A majority of transcripts showed similarity to Phaeophyceae (87%). The results of the BUSCO analysis revealed a near-complete gene sequence information for our transcriptome with 262 complete BUSCO matches (86.5%), 23 fragmented BUSCOs matches (7.6%) and 18 missing BUSCOs (5.9%). Additional information concerning transcriptome statistics and annotation results is available in Additional file 1.

**Fig. 1:**
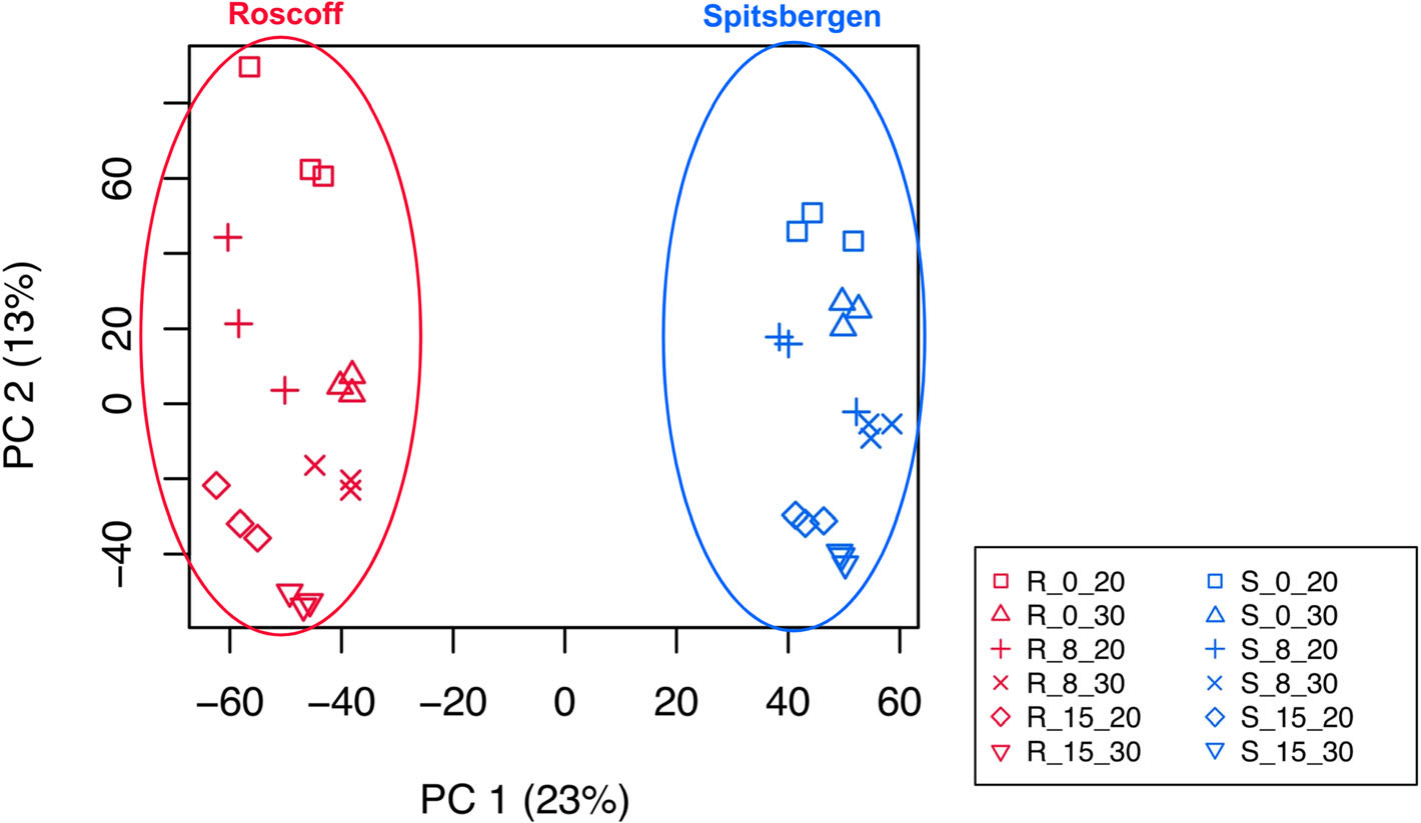
Principal Component Analysis of the counts per million in the treatments (*n* = 3)

Our results from Principal Component Analysis (PCA) of counts per million showed that the replicates of the treatments clustered well (Fig. 1). The first axis (PC1) explained 23% of the variability and clearly differentiated between sporophytes originating in Roscoff and Spitsbergen. The second axis (PC2) explained 13% of the variability and differentiated between salinity x temperature treatments. Spitsbergen samples were separated by temperature (0 °C > 8 °C > 15 °C). Roscoff specimens from 0 °C and 8 °C first grouped by low salinity, then by control salinity and finally all 15 °C samples (both low and control salinity) grouped together.

### Differential expression of Spitsbergen vs. Roscoff sporophytes at control conditions (8_30)

To investigate differences between the responses of Arctic versus temperate sporophytes suggested by the PCA (Fig. 1), we first looked at differences in gene expression between samples of Spitsbergen and Roscoff under control conditions (8_30): 958 differentially expressed genes (DEGs) (120 annotated with the UniProt Swiss-Prot database) were down-regulated in Roscoff in comparison to Spitsbergen material while 1766 (162 annotated) were up-regulated (Additional file 2).

We identified DEGs involved in nucleic acid metabolism, transport, cell wall synthesis and reorganization, general stress response, signaling and amino acid metabolism. In addition, nine genes involved in lipid metabolism were up-regulated in Roscoff control samples in contrast to only 2 genes in the Spitsbergen specimens (Additional file 2).

Several genes with cell wall-associated functions were differentially expressed between the control samples from the two locations. Namely, genes reported in higher plants such as callose synthases were up-regulated in Spitsbergen samples and UDP-D-xylose: L-fucose alpha-1,3-D-xylosyltransferase was up-regulated in Roscoff samples with a log_2_-fold change of 6.3 among others. Moreover, genes with known cell wall-associated functions of brown seaweeds were found. Four transcripts of vanadium-dependent bromoperoxidase were up-regulated in Roscoff and two were up-regulated in Spitsbergen samples. In addition, a mannuronate C5-epimerase was up-regulated in Roscoff and two in the Spitsbergen control samples (Additional file 2).

### Differential expression – salinity/temperature treatments compared to the control of algae from each geographical origin

**Fig. 2:**
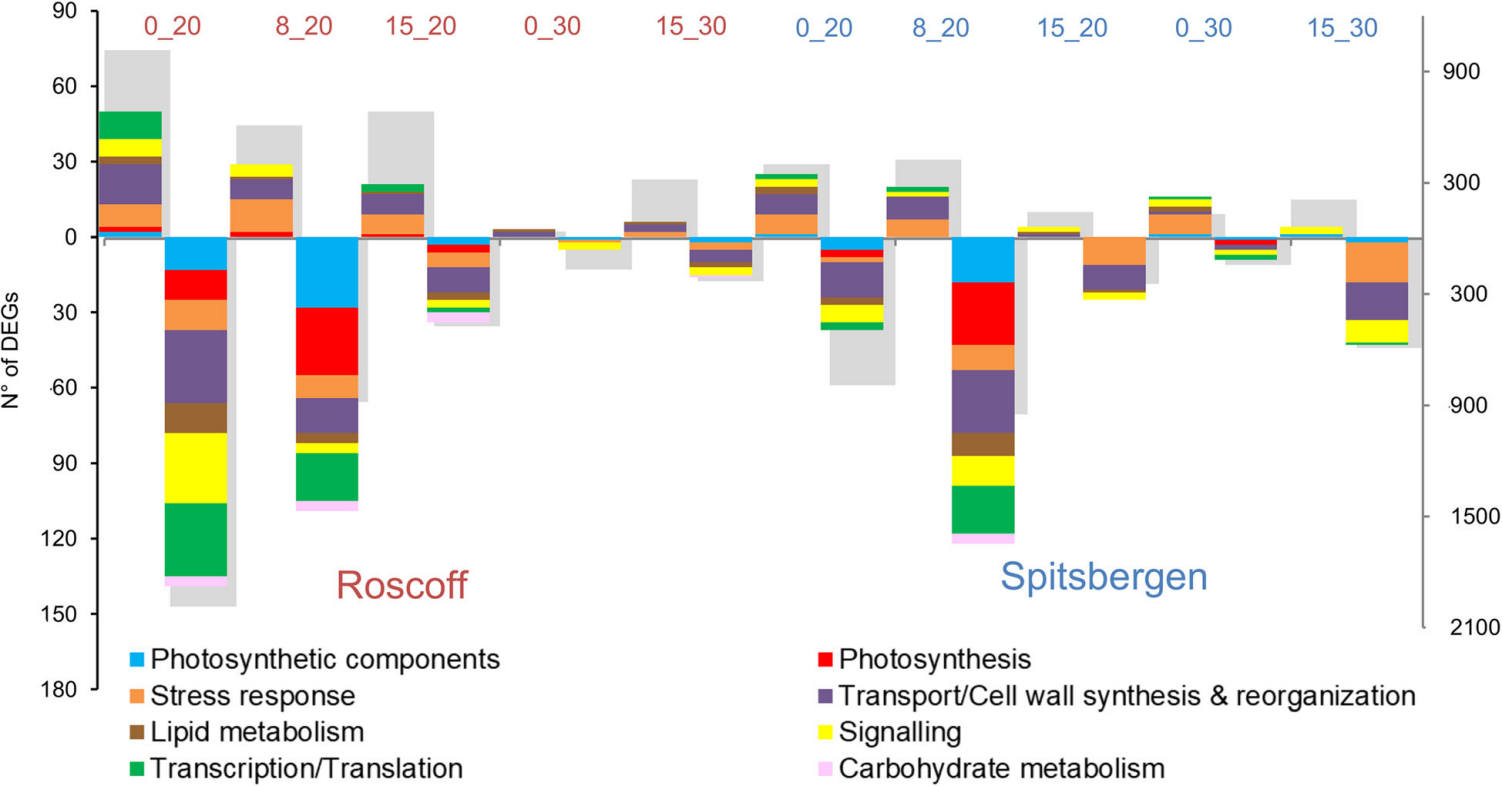
Differentially expressed genes in response to temperature and salinity by geographical origin. On the right axis, total number of significantly up- and down-regulated DEGs compared to the control (8_30) of sporophytes from each location (*p* < 0.001; log_2_FC > 2) is presented in grey bars. On the left axis and in colored bars number of DEGs compared to the control (8_30) according to functional categories of interest. Upper part of the graph displays up-regulated DEGs; the lower part down-regulated DEGs

Compared with the control (8_30) 4610 unique DEGs were found in specimens from Roscoff and 2966 from Spitsbergen. In the algae originating in Roscoff, the R_0_20 treatment caused the highest number of DEGs (47% - 3003), followed by R_8_20 (23% - 1491), R_15_20 (18% - 1160), R_15_30 (9% - 549) and finally R_0_30 (3% - 203) (Fig. 2 and Additional file 3). The low temperature/low salinity treatment accounted for almost half of the differential gene expression in the Roscoff samples.

In the sporophytes from Spitsbergen the pattern was different: the S_8_20 treatment induced the largest differential expression 34% (1374), followed closely by S_0_20 (30%- 1193), then by S_15_30 (20% - 801), S_15_20 (10% - 384) and finally by S_0_30 (7% - 274) (Fig. 2 and Additional file 3). Temperature treatments alone accounted for 12% of DEGs in sporophytes from Roscoff and for 27% in sporophytes from Spitsbergen (Additional file 3).

**Fig. 3:**
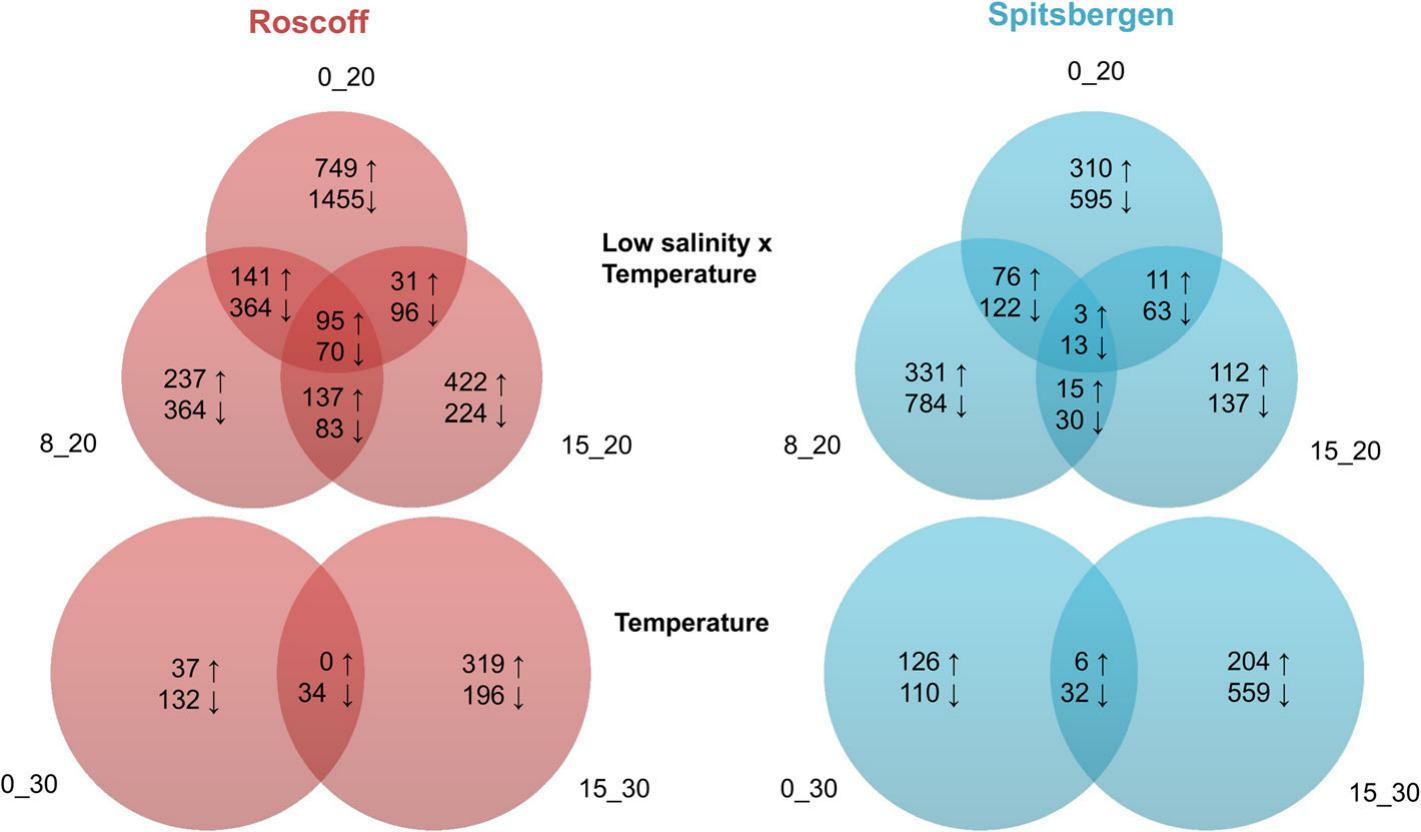
Venn diagrams of differentially up-regulated (↑) and down-regulated (↓) genes in the low salinity and in the temperature exposure treatments compared to the control (8_30) (p < 0.001; log_2_FC > 2)

The Venn diagrams show that for algae from each location only few genes overlapped between treatments (Fig. 3). Moreover, a reduced percentage of DEGs per treatment was shared among sporophytes from the two sites. It varied from 4% for up-regulated genes in 15_20 to 17% for down-regulated genes in 8_20 (Additional file 3).

### GO enrichment analysis

Several GO terms were enriched under the applied treatment conditions (Additional file 5): the numbers ranged from 44 (up-regulated DEGs in S_15_30) to 404 (down-regulated DEGs in S_8_20). Among up-regulated DEGs, the number of enriched GO terms was enhanced in R_8_20, R_15_20 and in R_15_30 compared to Spitsbergen samples, while at 0 °C more enriched GO terms were found in Spitsbergen (Additional file 5). The treatments resulted in a shift in several metabolic pathways such as lipid metabolism and nucleic acid metabolism, protein synthesis and modification, and in cell structure (Additional file 6). For the same treatment, some categories of enriched GO terms were only present in one of the location’ samples, e.g. aminoacid metabolism among down-regulated DEGs at S_0_20, apoptosis among up-regulated DEGs at S_15_30. Other categories showed GO terms in higher quantity in specimens of one geographical origin compared to the other, e.g. more GO terms within lipid metabolism in the Roscoff samples among up-regulated DEGs at 0_20; more GO terms within metabolism in the Spitsbergen specimens among down-regulated DEGs at 15_20 (Additional file 6). Among enriched GO terms in the up-regulated DEGs at 0_20 and 15_20, there were some prominent differences between sporophytes from the two geographical origins in the categories cell signaling/communication and carrier proteins/membrane transport. At 0_20 more enriched GO terms were found in the category cell signaling/communication in Roscoff than in Spitsbergen specimens while more enriched GO terms functioning within carrier proteins/membrane transport were found in Spitsbergen samples; at 15_20 the pattern was reversed (Additional file 6).

### Manual classification of DEGs

To identify the most important mechanisms involved in temperature and salinity acclimation and to investigate differences driven by geographical variation, we manually classified and examined all the annotated DEGs using the UniProt Swiss-Prot database as a starting point. The most obvious distinction was that in sporophytes from Roscoff more DEGs were regulated in several categories than the Spitsbergen isolates in response to the applied treatments.

Genes involved in transcription and/or translation were down-regulated at low salinity and decreased from R_0_20 (29 DEGs) to R_8_20 (19) to R_15_20 (2) but they were not differentially expressed in the temperature x control salinity treatments. In comparison, algae from Spitsbergen showed similar response at S_8_20 but less DEGs were regulated at S_0_20 (Fig. 2 and Additional file 7).

A large proportion of transcriptional regulation in the low salinity treatments was related to transport/ cell wall synthesis and reorganization (Fig. 2 and Additional file 7). In Roscoff, the highest number of DEGs was observed at R_0_20 (45), where the majority of genes were down-regulated (29) but 16 were up-regulated. While in Spitsbergen, the highest number of DEGs was promoted by S_8_20 (34) followed by S_0_20 (22); expression at 15 °C was similar in both salinity treatments (Fig. 2 and Additional file 7). A higher number of DEGs were identified within the class ‘signaling’ in Roscoff than in Spitsbergen samples, especially at 0_20 (Fig. 2 and Additional file 7).

#### Photosynthesis and pigment synthesis

In response to the hyposalinity treatments, a considerable number of DEGs were related to photosynthesis and photosynthetic components (93) in the algae from Roscoff, among these most were down-regulated (86). In comparison, in the control salinity treatments very few genes were differentially expressed in these categories (Fig. 2 and Additional file 7). However, in the samples from Spitsbergen, 43 DEGs were repressed at S_8_20, only 9 were expressed at S_0_20 and none at S_15_20 for the same categories. Similarly to Roscoff samples, very little transcriptional regulation was promoted by control salinity treatments (Fig. 2 and Additional file 7).

When comparing DEGs encoding transcripts related to photosynthesis between geographical origins, there were no noticeable differences in the magnitude of fold changes (Additional file 4). They were mostly down-regulated with a log_2_-fold change of around 2 and often triggered by the same treatments (Additional file 4). The most obvious difference was that several DEGs regulated in Roscoff were not observed in Spitsbergen individuals (Additional file 4). Namely, genes related to the light harvesting complex (e.g. chloroplastic chlorophyll a-b binding protein CP29.2, chloroplastic chlorophyll a-b binding protein 2) and electron transport (e.g. chloroplastic photosystem II 12 kDa extrinsic protein and chloroplastic oxygen-evolving enhancer protein 1) (Additional file 4).

Examples of genes with dynamic changes include magnesium chelatase subunit H, chloroplastic thioredoxin reductase NTRC (NTRC) and chloroplastic glutamate-1-semialdehyde 2,1-aminomutase (Additional file 4). Magnesium chelatase was down-regulated in all low salinity treatments in Roscoff samples but it was not regulated in Spitsbergen specimens (Additional file 4). Moreover, NTRC was down-regulated in R_0_20 samples and glutamate-1-semialdehyde 2,1-aminomutase was down-regulated in R_8_20, R_15_20 and in S_8_20 (Additional file 4). Four genes encoding chloroplastic pheophorbide *a* oxygenases were differentially expressed in our study. Two genes were induced in the R_0_20 and R_8_20 treatments; one of them was also induced in R_15_20. However, a third gene was repressed in R_0_20 and a fourth one in the S_8_20 sporophytes (Additional file 4).

Concerning the violaxanthin cycle, chloroplastic violaxanthin de-epoxidase was down-regulated in the S_8_20, R_0_20 and R_8_20 treatments while chloroplastic zeaxanthin-epoxidase was repressed in S_8_20 and R_0_20 specimens (Additional file 4).

Regarding the results of pigment analysis (additional file 8), violaxanthin had a significantly higher concentration in R_0_20 than in R_0_30 (*p* = 0.010, pairwise comparisons, Bonferroni adjustment), but no differences were found for 8 °C. In Spitsbergen samples, violaxanthin contents were significantly higher in the low salinity treatments than in the control (*p* = 0.025). The zeaxanthin content did not significantly change in Roscoff samples, however in specimens from Spitsbergen, zeaxanthin concentration at 0 °C was significantly higher than at 15 °C (*p* = 0.031, pairwise comparisons, Bonferroni adjustment).

#### Carbon metabolism

Concomitantly with repression of genes important for photosynthesis, several enzymes related to carbon metabolism were down-regulated under hyposalinity. Namely, chloroplastic phosphoribulokinase and chloroplastic fructose-1,6-bisphosphatase were down-regulated in R_8_20 samples while transketolase, cytosolic fructose-bisphosphate aldolase and cytosolic glucose 6-phosphate isomerase were repressed under the same conditions in Spitsbergen samples. Furthermore, chloroplastic ribulose-1,5 bisphosphate carboxylase/oxygenase large subunit N-methyltransferase was repressed at 8_20 in Spitsbergen and Roscoff specimens (Additional file 4). Moreover, cytosolic glyceraldehyde-3-phosphate dehydrogenase (GAPDH) was down-regulated at R_15_20 but up-regulated at 0 °C and 15_30 in Spitsbergen specimens (Additional file 4).

#### Stress responses

In Roscoff samples, a higher number of DEGs involved in oxidative stress response stress were expressed in the low salinity compared to the control salinity treatments. However, there was a mixed response with both induction and repression of gene expression (Fig. 2 and Additional file 7). Some reactive oxygen species (ROS) related genes were down-regulated, e.g. chloroplastic thioredoxin reductase NTRC (NTRC) in R_0_20 and heme-binding peroxidase at all low salinity treatments (Additional file 4). The response of the latter might be explained by the fact that it was already constitutively expressed in the control 8_30. Heme-binding peroxidase expression level in the control samples (Roscoff - 98.7 transcripts per kilobase million (TPM); Spitsbergen - 217 TPM) was higher than the average of all expressed genes (Roscoff - 21.6 TPM; Spitsbergen - 21.6 TPM) (Additional file 9). Nearly no ROS scavenging related genes were up-regulated in R_0_30 sporophytes, whereas Spitsbergen material showed some induction (Fig. 2 and Additional file 7). On the other hand, more ROS related DEGs were induced at 0_20 and less at 15 °C in Roscoff samples compared to Spitsbergen material (Fig. 2 and Additional file 7).

Superoxide dismutase (Fe) was down-regulated in S_8_20, R_8_20 and R_15_20 specimens, a cytosolic L-ascorbate peroxidase (APX) was down-regulated in S_8_20, R_0_20 samples, furthermore a chloroplastic APX was up-regulated in R_8_20 (Additional file 4). Two alternative oxidases were differentially expressed in our setup. The chloroplastic ubiquinol oxidase was induced at 0 °C in Spitsbergen samples and also at R_0_20 while it was repressed at 15 °C in Roscoff specimens and the alternative oxidase (mitochondrial) was up-regulated at 0_20 in both Roscoff and Spitsbergen samples (Additional file 4). A peptide methionine sulfoxide reductase (MsrA) gene was up-regulated in response to all low salinity treatments in Roscoff samples and in S_0_20 treatments, however another gene was down-regulated in S_8_20 (Additional file 4). The chloroplastic lactoylglutathione lyase was repressed in R_0_20, R_8_20 and S_8_20 samples (Additional file 4). Glutathione S-transferases (GSTs) were induced in R_0_20 and R_8_20 and repressed in 15 °C (around − 2 log_2_FC) samples (Additional file 4). In Spitsbergen samples, GSTs showed the same pattern with the exception that in the 0_30 treatment they were up-regulated in Spitsbergen and showed no changes in sporophytes from Roscoff (Additional file 4). Twenty genes encoding vanadium-dependent bromoperoxidases were differentially expressed in our experiment. They were mostly down-regulated in R_0_20 and R_8_20 and up-regulated at 15 °C in Roscoff samples. However, in the Spitsbergen samples, they were induced at S_8_20 and repressed in the 15 °C treatments. Moreover, we did not find any vanadium-dependent iodoperoxidases (Additional file 4). When looking at the TPM, we identified nine antioxidant genes constitutively expressed in the control from both locations and three were expressed at highly different levels between Roscoff and Spitsbergen samples – two (chloroplastic thioredoxin-like protein HCF164 and glutaredoxin-C2) presented higher expression in Roscoff than in the Spitsbergen samples while the reverse was observed in another one (heme-binding peroxidase) (Additional file 9). Concerning chaperones, HSPB1-associated protein 1 was repressed in R_0_20 while 10 kDa chaperonin was repressed in R_8_20 samples. Chaperone protein ClpB 2 was repressed in S_8_20 and the 78 kDa glucose-regulated protein was repressed in S_15_30 samples (Additional file 4). Furthermore, we identified sixteen chaperones constitutively expressed in the control of Roscoff and Spitsbergen samples and three chaperones with clearly different expression profiles between Roscoff and Spitsbergen samples - two at higher expression levels in the Spitsbergen samples (heat shock 7 kDa protein 5 and heat shock protein 9–1), one was expressed at higher levels in the Roscoff ones (assembly chaperone of rpl4) (Additional file 9).

#### Salinity specific response

Mannitol 2-dehydrogenase was among the expressed genes but it was not significantly differentially expressed. No other genes related to mannitol biosynthesis were found. The lack of differential expression may be the result of constitutive expression in the control specimens (Additional file 9). Proline dehydrogenase 1 (mitochondrial) was also among the expressed genes but it was not differentially expressed. Moreover, its expression level, in TPM, was below the average.

#### Glycerophospholipid metabolism

We detected three desaturases down-regulated in low salinity treatments (Additional file 4). Stearoyl-CoA desaturase 5 was down-regulated at 0_20 in Roscoff and Spitsbergen samples, sn-2 acyl-lipid omega-3 desaturase (FAD7) was down-regulated at S_8_20, R_0_20 and R_8_20 and a delta-fatty-acid desaturase (FAD2) was down-regulated at R_0_20 samples. A choline kinase was down-regulated in S_8_20, R_0_20 and R_8_20 sporophytes. One gene encoding oxygen-dependent choline dehydrogenase was down-regulated at S_0_20 and S_8_20. Another gene was repressed at 0 °C and S_8_20 in Spitsbergen, R_0_20 and R_8_20 samples (Additional file 4).

## Discussion

Out of the 6114 DEGs in our study (unique DEGs versus control in specimens from both locations), only 756 (12.4%) could be functionally annotated using the UniProt Swiss-Prot database. Therefore, almost 90% of the genes regulated in *S. latissima* under a combination of temperature and salinity stress remain unknown. These results highlight the potential and need to discover new metabolic pathways required by the species to tackle environmental changes and further support brown algae as a relevant piece in understanding evolutionary history [25].

The number of DEGs in our study is comparable to the numbers reported in other studies, i.e. in *Desmarestia anceps* in response to light, temperature and CO_2_ [between 337 and 3255 DEGs in the comparison to the control [26];].

### Transcriptomic differences driven by geographical variation

The response of sporophytes from Arctic Spitsbergen and temperate Roscoff differed in many aspects. These differences became apparent during all applied analyses (PCA, differential expression and GO enrichment). A higher number of DEGs was found in Roscoff specimens than in the Spitsbergen ones when comparing treatments against the respective control. In addition, there was little overlap between DEGs under the same exposure conditions in the sporophytes originating in distinct geographical sites. These results and the extensive disparity among GO enrichments demonstrate that not only the magnitude of the response differs between specimens of the two locations but also that the metabolic reorganization was performed differently. Moreover, several genes were differentially expressed between the control samples of Spitsbergen and Roscoff, even though they were cultured under the same conditions.

Physical properties of the cell might be affected under changing ambient conditions [27, 28]. DEGs with cell wall and cytoskeleton-associated functions, which are critical in the cell’s adjustment to temperature and osmotic fluctuations, were observed. An example of a DEG between the control samples is callose synthase that catalyzes the synthesis of callose in plants [29]. Callose regulates permeability in plasmodesmata, which play a role in cell to cell communication and transport of macromolecules. Callose accumulation is affected both by developmental cues and stress in plants [30]. Recently, an immunolocalization study found a callose (β-(1,3)-glucan) epitope in the cell walls of the brown alga *Fucus vesiculosus* and in the sieve plates of *Laminaria digitata* [31]. Furthermore, cell wall adjustment was identified as one of the main processes in acclimation to freshwater in an *Ectocarpus siliculosus* sister species [32]. The same study also connected changes in transcripts such as mannuronate C5-epimerase and vanadium dependent bromoperoxidases (V-BPOs) with differences in morphology between the sister species [32]. This is comparable to our study in *S. latissima*, as sporophytes used in our experiment had different phenotypes since an early stage of cultivation (Additional file 10). Sporophytes originating in Spitsbergen were more elongated and narrower than sporophytes from Roscoff and attained a lower fresh weight after three months of cultivation. In the comparison of exposure treatments to the respective controls, V-BPOs were mostly down-regulated at 0 °C and 8 °C low salinity and up-regulated at 15 °C in the Roscoff samples. However, in algae from Spitsbergen, they showed a contrasting pattern – induction at 8 °C low salinity and repression at 15 °C. Contrary to our data, V-BPOs did not change in response to hyposalinity stress in *S. japonica* [33] nor in *Ectocarpus siliculosus* [34], however they are involved in the response to other environmental stressors in kelps, e.g. in *Macrocystis pyrifera* [35] and in *Laminaria digitata* [36]. Furthermore, differential gene expression of V-BPOs has been previously identified in *S. latissima*: the interaction of temperature, light and UV-radiation resulted in a mixed response after two weeks exposure, however, V-BPOs were mostly up-regulated at higher temperatures [16] and down-regulated in response to low temperatures after 24 h [15]. Regulation of V-BPOs in our study might be connected to cell wall adjustments in response to osmotic stress as V-BPOs have been reported to promote the binding of phenolic substances to alginates in algal cell walls [37].

Moreover, we found several genes associated with lipid metabolism, mostly induced in Roscoff control samples. Changes in lipids within membranes will affect its composition, integrity and fluidity [38]. Altogether, our results indicate that cell wall and membrane adjustment might be important mechanisms in the acclimation of *S. latissima* to contrasting environmental conditions.

Differences in treatment responses driven by geographical origin included the expression of transcripts encoding photosynthesis related components. Although photosynthesis was repressed under low salinity in samples from both locations, more DEGs were down-regulated in specimens from Roscoff than from Spitsbergen. Additionally, a key enzyme in chlorophyll catabolism, pheophorbide *a* oxygenase [39], was induced in Roscoff samples but repressed in Spitsbergen material. Also, magnesium chelatase and NTRC, which play key roles in the regulation of chlorophyll metabolism [40] were only differentially expressed in Roscoff samples. This suggests that hyposalinity stress was more severe for sporophytes originating in Roscoff than in Spitsbergen, demanding a stronger reduction in photosynthesis. This might be explained by the fact that in Roscoff algae are exposed to a stable saline environment and are less able to adjust to salinity changes. Moreover, at lower temperatures photo-inhibition of photosynthesis may occur [41], a process that might be more frequent in algae seldom exposed to cold temperatures, such as in specimens from Roscoff.

Furthermore, both GO enrichment and DEGs results showed enhanced cell signaling at 0_20 in Roscoff samples compared to Spitsbergen and at 15 °C in Spitsbergen compared to Roscoff material. This pattern suggests that the low temperature and low salinity conditions tested are not commonly encountered in Roscoff and therefore required a stronger signaling response than in Spitsbergen samples in order to trigger physiological adjustments. Differential expression of signaling related genes as a result of local adaptation has so far, to our knowledge, not been described in algae. However, in the marine realm, it has been observed between closely related species of mussels with distinct heat tolerance [42] and between populations of a species of killifish in response to local salinity variability [43].

### Responses to salinity and temperature changes

Several metabolic pathways were involved in the response to temperature and salinity variation in *S. latissima*, demonstrating the relevance of these factors to its physiology and ecology. To capture the responses to relevant ecological variation at the sites, the levels of the applied stress corresponded to levels experienced at these sites (Roscoff [23], Spitsbergen [22]) and fall well within the physiological limits of the species [10–13]. Thus, the applied stress was only mild, which might explain why some classic stress responses were not observed, such as strong up-regulation of heat shock proteins, antioxidant enzymes and catabolic processes, as previously observed in *S. latissima* in response to high light and high temperature [16] and in several other macroalgae in response to severe stress levels [44–46]. Still, our results help to unravel how acclimation to temperature and salinity changes is achieved in *S. latissima*. Pathways involved included signaling, photosynthesis and pigment synthesis, stress related enzymes, transport and cell wall synthesis and reorganization.

The analysis of DEGs shows that the low salinity treatments had a higher impact on the number of genes regulated than temperature alone in sporophytes from both locations; however, the proportion and the magnitude of impact were different between treatments. Given that the algae had already been exposed for eight days to the experimental temperatures before the onset of the salinity treatment, the limited response to temperature may indicate that the sporophytes were already acclimated [47] and observed differences are only the long-term changes in gene expression and not short-term stress responses. In turn, salinity driven responses were only evaluated after 24 h and therefore the differential impact of temperature and salinity on gene expression profiles might be driven by temporal variation in responses. As we assessed only one sampling point, we cannot infer about the temporal dynamics of the response and further studies are necessary to understand the regulatory kinetics in the acclimation to these abiotic factors.

Only a few DEGs overlapped between treatments applied to algae from the two locations. This indicates that a common response to salinity or temperature stress was not evident, but interactions between the two factors were apparent. The higher temperature (15 °C) triggered a smaller response than 0 °C, especially in the Roscoff sporophytes.

We observed down-regulation of photosynthesis and carbon assimilation which seems to be a common response pattern to salinity changes as it was also found in a diatom, another brown alga and terrestrial plants [34, 48, 49]. Additionally, we observed down-regulation of enzymes contributing to the xanthophyll cycle at S_8_20 and R_0_20. However, we were not able to link gene expression with the pigment content measured in our samples (Additional file 8). The xanthophyll cycle is an important photoprotection mechanism in vascular plants, green and brown seaweeds, and related to the de-epoxidation of violaxanthin to zeaxanthin, leading to dissipation of excess excitation energy [50]. In our study, down-regulation of xanthophyll cycle enzymes might have been connected to a reduced photosynthetic capacity thus reducing the need for photoprotection. The dark operation of the xanthophyll cycle has been observed in response to abiotic stressors in another brown alga, *Pelvetia canaliculata* [51]. However, to the best of our knowledge, our study is the first potentially implicating the xanthophyll cycle in salinity stress responses.

Heat shock proteins (HSPs) are often induced under abiotic stress [52]. Previously, 13 genes encoding HSPs were reported to be differentially expressed in *S. latissima* under temperature and light stress after 24 h [15]. Unexpectedly, we only found four HSPs DEGs regulated after eight days of acclimation to temperature (0 °C and 15 °C). This indicates that HSPs are rather induced in short-term responses but reduced in longer term acclimation, which was also shown by Heinrich et al. [16]. Alternatively, it suggests that HSPs are not intensively involved in response to cold, as already reviewed in Sørensen and Loeschcke [53], and that the highest temperature tested, 15 °C, was not high enough to induce HSPs. This hypothesis is supported by reports on a stronger transcriptional regulation of HSPs in response to high (17 °C) than to low temperature (2 °C) [15]. Moreover, Dittami and colleagues found three HSPs, one down-regulated and two up-regulated in a hyposalinity treatment in *E. siliculosus* [34]. Taken together, these results suggest that HSP regulation in response to salinity variation takes place to a lesser extent than in response to temperature.

Genes involved in reactive oxygen metabolism often play a key role in stress responses [54, 55]. The main ROS scavenging enzymes in plants are superoxide dismutase (SOD), ascorbate peroxidase (APX) and catalase (CAT) [55]. However, those did not seem to be involved in the response to stress in our study as they were either down-regulated (SOD, APX) or not regulated (CAT). Lactoylglutathione lyase, that catalyzes the synthesis of methylglyoxal, was also repressed under hyposalinity (R_0_20, R_8_20 and S_8_20). The reduction in methylglyoxal production might be a stress tolerance strategy as was already reported in plants [56] and in the kelp *Lessonia spicata* in response to desiccation [57].

On the other hand, some stress related genes were induced in response to the temperature and salinity levels tested in our experiment. Alternative oxidases can reduce ROS production by diverting electrons from the electron transport chains to produce water from oxygen [55]. Two alternative oxidases were mostly up-regulated in our study. One gene encoding an ubiquinol oxidase was up-regulated at 0 °C in Spitsbergen specimens, up-regulated in Roscoff sporophytes at 0_20, but down-regulated at 15 °C. A second gene was also up-regulated at 0 °C in Spitsbergen samples. Furthermore, peptide methionine sulfoxide reductase has an important function as a repair enzyme for proteins that have been inactivated by oxidation [58]. It was also shown to be up-regulated in *Laminaria digitata* in response to biotic stress [59]. The low salinity treatments provoked up-regulation of MsrA - at 0_20 in Spitsbergen samples, and at all low salinity treatments in Roscoff samples, indicating that Spitsbergen specimens were less affected by low salinity.

Glutathione S-transferases are enzymes that conjugate glutathione with electrophilic components, playing several roles in eukaryotic cells such as detoxification of xenobiotics, metabolism of aromatic amino acids and lipids [60]. In algae, they have been measured in response to environmental pollution and to abiotic stress [36, 61]. In our data, GSTs were up-regulated at 0 °C and 8 °C and down-regulated at 15 °C in samples from both locations. Although GSTs play different roles in cell metabolism and therefore more studies are necessary to determine their exact function, they seem to be linked to temperature acclimation in the conditions of our experiment.

In our study, we failed to detect changes in transcripts coding for enzymes involved in the synthesis of the known osmolyte in brown algae, mannitol [62] and proline, with a similar function in plants and diatoms [63, 64]. This finding is contrary to previous results on the down-regulation of a mannitol 1-phosphate dehydrogenase and the induction of proline dehydrogenase in response to a hyposalinity treatment in *E. siliculosus* [34]. However, oxygen-dependent choline dehydrogenase was down-regulated at 0_20 and 8_20 in Roscoff and Spitsbergen’ sporophytes. This enzyme is involved in the biosynthesis of glycine betaine, an osmoprotectant in terrestrial plants [65]. However, to the best of our knowledge, there are no reports of similar functions in Phaeophyceae, even though the compound has been shown to be present in brown algae [66]. If this osmolyte would have a similar function in *S. latissima*, we would expect its biosynthesis to be induced. All together, we can speculate that *S. latissima* does not rely on synthesis of the osmolytes proline and mannitol during short-term responses to salinity. Metabolomics and proteomics studies would help us understand if this regulation is not part of the response or if it is post-translational.

Phospholipids are an important component of cell walls, and modifications of their composition leads to adjustment of membrane fluidity in response to environmental change [38, 67]. Choline kinases catalyze the first step in phosphatidylcholine biosynthesis, a phospholipid present in eukaryotic cell membranes [68]. Choline kinases have been suggested as key regulatory enzymes in salt stress response in *Arabidopsis* [69] and they were partially repressed in response to hyposalinity in our study - S_8_20, R_0_20 and at R_8_20. Moreover, we found three desaturases regulated in our experiment - FAD7, FAD2 and a stearoyl-CoA desaturase. Desaturases are enzymes that catalyze the biosynthesis of polyunsaturated fatty acids [67]. Changes in their expression in *Ectocarpus* were associated with adaptation to freshwater [32]. Moreover, the expression of FAD7 in transgenic tobacco increased cold tolerance in the plant by increasing levels of trienoic fatty acids [70]. An increase in unsaturation of fatty acids in winter has also been reported in polar macrophytes [71]. However, the desaturases genes differentially expressed during our study were down-regulated and restricted to the low salinity treatments (0_20 and 8_20).

The limited number of genes involved in known stress responses regulated in this study, namely HSP and proteins with antioxidant activity, are in accordance with responses of the brown alga *E. siliculosus* and the red alga *Chondrus crispus* to hyposalinity [34, 61]. A suggested explanation is that post-transcriptional regulation is a more important mechanism than transcriptional regulation modulating stress related proteins. This is supported by the identified mismatch between gene expression and protein data in *Arabidopsis* in response to salt stress [71]. Another reason might be that the strong down-regulation of photosynthesis, namely chlorophyll binding proteins, reduced the amount of energy reaching the photosynthetic reaction centers and therefore reduced the production of reactive oxygen species [72]. However, an alternative explanation is that some classic stress genes are already constitutively expressed in the control (Additional file 9) and given that we used a stringent threshold (*p* < 0.001, log_2_FC > 2) for our differential expression analysis, we failed to detect smaller variations. This constitutive expression may allow *S. latissima* to acclimate to stress quickly and might be part of its broad tolerance to stress.

### Ecological implications

The limited response to temperature changes in sporophytes irrespective of their origin suggests a high tolerance to temperature of *S. latissima*, which is mirrored in its broad latitudinal and vertical distribution [6, 7]. Although *S. latissima* is currently not exposed to seawater temperatures of 15 °C in the Arctic, this temperature did neither promote extensive transcriptomic changes nor stress responses. However, in comparison to the Roscoff specimens, it showed a larger overall response and indeed a higher number of antioxidant genes were regulated in Spitsbergen material at 15 °C.

Hyposalinity had a higher impact at the transcriptional level than temperature alone. This suggests that acclimation to this stressor might be more costly and therefore has a higher potential to impact growth at these locations. The interaction between low salinity and low temperature was especially stressful for Roscoff sporophytes and triggered an extensive repression of photosynthesis, with presumably great impact on physiology and growth of the specimens. On the other hand, higher temperatures appear to ameliorate the hyposalinity stress. This is the first study investigating transcriptomic responses to salinity in *S. latissima* and even in physiological studies this abiotic factor has been remarkably underrepresented. Given our results, to better understand current local physiological performance and its modulation by future global change, more research targeting salinity and its interactions with other factors is necessary.

## Conclusions

Resilience of macroalgae stands to climate change was identified as population-specific in some studies e.g .[73]. The use of the transcriptomic data to unravel local adaptation and/or phenotypic plasticity is a recent but promising strategy [42, 74, 75]. In brown algae, Jueterbock et al. [76] demonstrated that the expression of heat shock proteins was population-specific in *Fucus serratus*. Similarly Mota et al. [77] described differences between cold and warm edge populations of *Fucus vesiculosus* in photosynthetic efficiency and expression of heat shock proteins, and Ritter et al. [78] showed that two populations of *E. siliculosus* presented different responses to copper stress. However, in these studies real-time PCR was used, a technique that restricts the analysis to a few genes of interest. RNA-sequencing allows uncovering large-scale transcriptomic responses without being limited to known sequences, and thus is a very powerful strategy especially in non-model organisms with limited genomic information. However, given the relatively high costs of this technology, experiments combining several factors and biological replicates are nearly unattainable. A previous study in *S. latissima* from Spitsbergen revealed that field and culture material responses to UV-radiation under different temperatures involved similar processes, even though in different intensities [17]. Therefore studies such as ours may provide the basis to select the abiotic factors and populations most likely contributing to the understanding of acclimation mechanisms in brown algae and giving insights into the modulation of responses along geographical gradients. We provide a large-scale transcriptomic analysis of temperature and salinity acclimation mechanisms in sporophytes originating in the central and northern distribution of the sugar kelp, *Saccharina latissima*. The differences between transcriptional responses of cultured sporophytes from the two locations to stress fit to a certain extent with our initial hypothesis that Roscoff sporophytes would perform better at higher temperatures and be affected more intensively by hyposalinity and that in turn Spitsbergen sporophytes would perform better at lower temperatures and at low salinities. The high number of DEGs reported between the respective controls together with their distinctive morphology even after cultivation under the same conditions further support that responses in these two geographical origins have diverged and that they might have adapted to their local conditions. The temperature tolerance of the Spitsbergen uniparental sporophytes observed here might confer resilience to a warming Arctic. Moreover, its better performance under hyposalinity conditions might be an advantage over the Roscoff sporophytes in case of a poleward shift of the latter. To better understand population transcriptomics in *S. latissima* future studies should include experiments with specimens originating in other sites in other populations, ideally from both field and cultivated material, and more genetic variability from the same locality is needed. Especially, information about populations living closer to their physiological limits would further elucidate the mechanisms underlying the broad tolerance and therefore broad distribution of the species.

## Methods

### Algal material

Young sporophytes of *S. latissima* were raised from stock cultures of uniparental male and female gametophytes at the Alfred-Wegener-Institute Helmholtz Centre for Polar and Marine Research (AWI, Bremerhaven, Germany; stock culture 3425 and 3426 Roscoff; 3123 and 3124 Spitsbergen) according to the protocol of Heinrich et al. [15]. The parental sporophyte material from which unialgal clonal gametophyte cultures were isolated [79] was collected in the wild and was identified by Andreas Wagner (Roscoff, Brittany, France; 48° 43′ 39″ N, 3° 59′ 13.2″ W) and by Christian Wiencke (Spitsbergen, Norway; 79°N, 11°E). *Saccharina latissima* is a kelp species distributed along the European coastline and easily and unmistakably to identify. No voucher specimens of the sporophytes were created. As material from Spitsbergen and Roscoff was collected in 1991 and 2013, respectively, it does not fall under the Nagoya legislation which regulates the access and benefit sharing (ABS) of biological resources since 12 October 2014. Sporophytes of both locations were grown aerated in glass beakers at 8 °C under a photon fluence rate of 20 μmol photons m^− 2^ s^− 1^ of photosynthetically active radiation (PAR) (Mitras Lightbar Daylight 150, GHL, Germany) with a 18 h light: 6 h dark photoperiod. Algae were cultivated for three months in sterile seawater enriched with Provasoli [80] with an absolute salinity (S_A_) [81] of ~ 30 until they reached an average fresh weight of 0.13 g (7–9 cm) for Spitsbergen sporophytes and 0.58 g (5–7 cm) for Roscoff sporophytes.

### Experimental set-up

At the start of the experiment, sporophytes were kept at the respective experimental temperature (0 °C, 8 °C and 15 °C) in temperature controlled rooms. Temperatures were chosen to mirror local conditions at sampling sites (Spitsbergen: 0 °C to 8 °C [22], Roscoff: 9 °C to 15 °C [23]) and to allow for a balanced experimental design where low temperature and high temperature treatments vary equally (+/− 1 °C) from the control temperature (8 °C). Control conditions were also selected with respect to optimal growth requirements of *S. latissima* [10, 11]. After one week, per each temperature, sporophytes were divided into a low salinity treatment of S_A_ 20 or kept under the control salinity (S_A_ 30). S_A_ 20 seawater was obtained by adding deionized water. We set the hyposalinity stress level at S_A_ 20 to guarantee that it was within tolerance levels [12, 13] and to represent an occasionally ecological relevant level in Spitsbergen following snow and ice melt run-off [20].

Each treatment was applied to 5 replicate aerated beakers (5 L) with 12 sporophytes each. After 24 h, whole sporophytes for RNA extraction (*n* = 3) and pigments quantification (*n* = 5) were taken. Sporophytes were frozen in liquid nitrogen and stored at − 80 °C until further use. Remaining sporophytes were stored for further analysis not covered in this manuscript.

### Pigment quantification and statistical analysis

Pigment analysis was performed according to the method described in Bollen et al. [82]. Then we calculated the percentage of initial values (before salinity acclimation) of violaxanthin and zeaxanthin concentrations. All data were tested for normality using the Shapiro-Wilk normality test and for homogeneity of variances using the Levene’s test. Similarity of pigment contents were tested using a two-way ANOVA with the fixed factors temperature and salinity. Significant differences and interaction of means were compared with the post hoc Tukey test (HSD). When data failed to comply with normality and homogeneity of variances assumptions, we performed a non-parametric test (Mann-Whitney U or Kruskal-Wallis test). All statistical analyses were carried out using SPSS software version 24 (IBM, Armonk, USA). The significance level for all analyses was set at α = 0.05.

### RNA-sequencing and data processing

#### Data obtained, quality control and trimming

Total RNA extraction was conducted using the method described in Heinrich et al. [83]. RNA quality was analysed by the NanoDrop ND-1000 UV-Vis Spectrophotometer and Agilent 2100 Bioanalyzer (Agilent Technologies, Germany). cDNA libraries were prepared with an Illumina TruSeq RNA Library Prep Kit according to the manufacturer protocol and sequenced in triplicates. The libraries were sequenced on an Illumina Hiseq 2500 and 75 bp paired reads were clipped using default values of the Illumina software.

Raw reads were quality controlled by FastQC v. 0.11.5 [84] and quality filtered using Trimmomatic v. 0.36 [85]. Quality filtering was performed using the following parameters: leading 3, trailing 3, sliding window 4:15, minlen 30.

#### Assembly

Reads from all treatments were assembled de novo all together and separately for sporophytes from Spitsbergen and Roscoff. The assemblies were performed with Trinity v 2.4.0 including the reads normalization step corresponding to the Trinity implementation of the diginorm method. The assembler was run with default parameters [86]. The quality of the transcriptome assemblies was evaluated by using BUSCO v2.0 [87] with eukaryote dataset (OrthoDB v9.1]. To check for potential contamination, bacterial and oomycete sequences obtained from Genbank were compared to the transcriptome assembly by sequence similarity search (blastn).

#### Mapping and DEG analysis

The reads from both Roscoff and Spitsbergen samples were pseudo-aligned with Salmon [88] against the three de novo assembled transcriptomes based on both libraries, Roscoff cDNA library only and Spitsbergen cDNA library only. A PCA plot of the counts-per-million, followed by a log_2_ transformation, of all treatments was generated by a Trinity script. Differential expression was calculated using DESeq2 [89] at Trinity’s gene level with an adjusted level of *P* ≤ 0.001 and a log_2_-fold change of at least 2 indicating significance. One differential expression analysis was performed comparing the control samples of Spitsbergen to the control samples of Roscoff. A second analysis compared each exposure treatment to the control of sporophytes from each location. The combination of three temperatures – 0 °C (0), 8 °C (8) and 15 °C (15) and two salinities – S_A_ 20 (20) and S_A_ 30 (30) resulted in five treatments compared to the control (8_30) per site of origin in a total of 10 comparisons (Spitsbergen (S): S_0_20, S_0_30, S_8_20, S_15_20, S_15_30; Roscoff (R): R_0_20, R_0_30, R_8_20, R_15_20, R_15_30).

Tools were executed using the scripts included in the Trinity package v 2.4.0 [86].

#### Functional annotation and enrichment

Functional annotation was performed using the Trinotate functional annotation pipeline [90] with the UniRef90 database as additional reference to the default database Uniprot Swiss-Prot database (all databases up to date in October 2017). To investigate the function of significantly up- and down-regulated genes, Gene Ontology (GO) enrichments were conducted using GOseq [91]. Enriched GO terms were summarized with CateGOrizer using the EGAD2GO classification file [92]. For exploring constitutively expressed transcripts within the control, normalized read counts, given as transcripts per kilobase million (TPM), were analysed, following the approach of Iñiguez et al. [26]. Venn diagrams were produced through a webtool [93].

## Supplementary information

**Additional file 1.** Summary of transcriptome statistics and of transcript annotations in the public databases.

**Additional file 2 **List of DEGs found in the comparison of control (8_30) between Spitsbergen and Roscoff samples; *P* ≤ 0.001 and log_2_-fold change > 2.

**Additional file 3.** Number of DEGs for each pairwise comparison per location; P ≤ 0.001 and log_2_-fold change > 2; Percentage of DEGs shared between Roscoff and Spitsbergen samples per treatment.

**Additional file 4.** List of DEGs found in the exposure treatments against control (8_30) of each location; P ≤ 0.001 and log_2_-fold change > 2.

**Additional file 5.** Number of significantly enriched GO terms among DEGs. Comparison of exposure treatments against control (8_30) of each location.

**Additional file 6.** Functional categories derived from enriched GO terms of differentially expressed genes of the treatments compared to the control (8_30): A) 0 °C treatments, B) 8 °C treatments, C) 15 °C treatments; sporophytes from Spitsbergen are represented in the blue bars and specimens from Roscoff in the red bars. Classification after EGAD2GO using cateGOrizer.

**Additional file 7.** Manual classification of DEGs compared to the control (8_30) according to functional categories of interest. ↑ indicates up-regulated DEGs, ↓ down-regulated DEGs.

**Additional file 8 **Results of the two-way ANOVA and graphic representation for effects of temperature, salinity and their interaction on the pigments measured after 24 h of temperature and salinity exposure. Statistically significant values are indicated by asterisks (*P* < 0.05). When data failed to comply with normality and homogeneity of variances assumptions, results of a non-parametric test (Mann-Whitney U or Kruskal-Wallis test) are showed.

**Additional file 9.** Transcript per million (TPM) counts of the control treatment (8_30) of sporophytes from each geographical origin corresponding to genes encoding chaperones, antioxidant proteins and salinity stress expected genes. In bold TPM values of genes considered constitutively expressed in the control, in red genes with TPM values considered highly different between samples from the two locations.

**Additional file 10.** Phenotypic differences between sporophytes from Roscoff and Spitsbergen before the start of the experiment. A) Spitsbergen, B) Roscoff.

## Data Availability

The Illumina sequence reads generated during the current study have been deposited in the Array express repository [94], under the accession number E-MTAB-7348.

## Abbreviations

APX: L-ascorbate peroxidase
CAT: Catalase
DEGs: Differentially expressed genes
FAD2: Delta-fatty-acid desaturase
FAD7: Sn-2 acyl-lipid omega-3 desaturase
GAPDH: Glyceraldehyde-3-phosphate dehydrogenase
GO: Gene ontology
GSTs: Gluthathione S-transferases
HSPs: Heat shock proteins
MsrA: Peptide methionine sulfoxide reductase
NTRC: Thioredoxin reductase NTRC
PAR: Photosynthetically active radiation
PCA: Principal component analysis
ROS: Reactive oxygen species
S_A_: Absolute salinity
SOD: Superoxide dismutase
TPM: Transcripts per kilobase million
V-BPOs: vanadium dependent bromoperoxidases
