# Supplementary material for: Is geographical variation driving the transcriptomic responses to multiple stressors in the kelp Saccharina latissima?
Source: BMC Plant Biol. 2019 Nov 21;19:513. doi: 10.1186/s12870-019-2124-0 (PMC6881991; doi:10.1186/s12870-019-2124-0)
Supplement: Supplementary file 6 — Additional file 6. Functional categories derived from enriched GO terms of differentially expressed genes of the treatments compared to the control (8_30): A) 0 °C treatments, B) 8 °C treatments, C) 15 °C treatments; sporophytes from Spitsbergen are represented in the blue bars and specimens from Roscoff in the red bars. Classification after EGAD2GO using cateGOrizer. [file 12870_2019_2124_MOESM6_ESM.pdf]

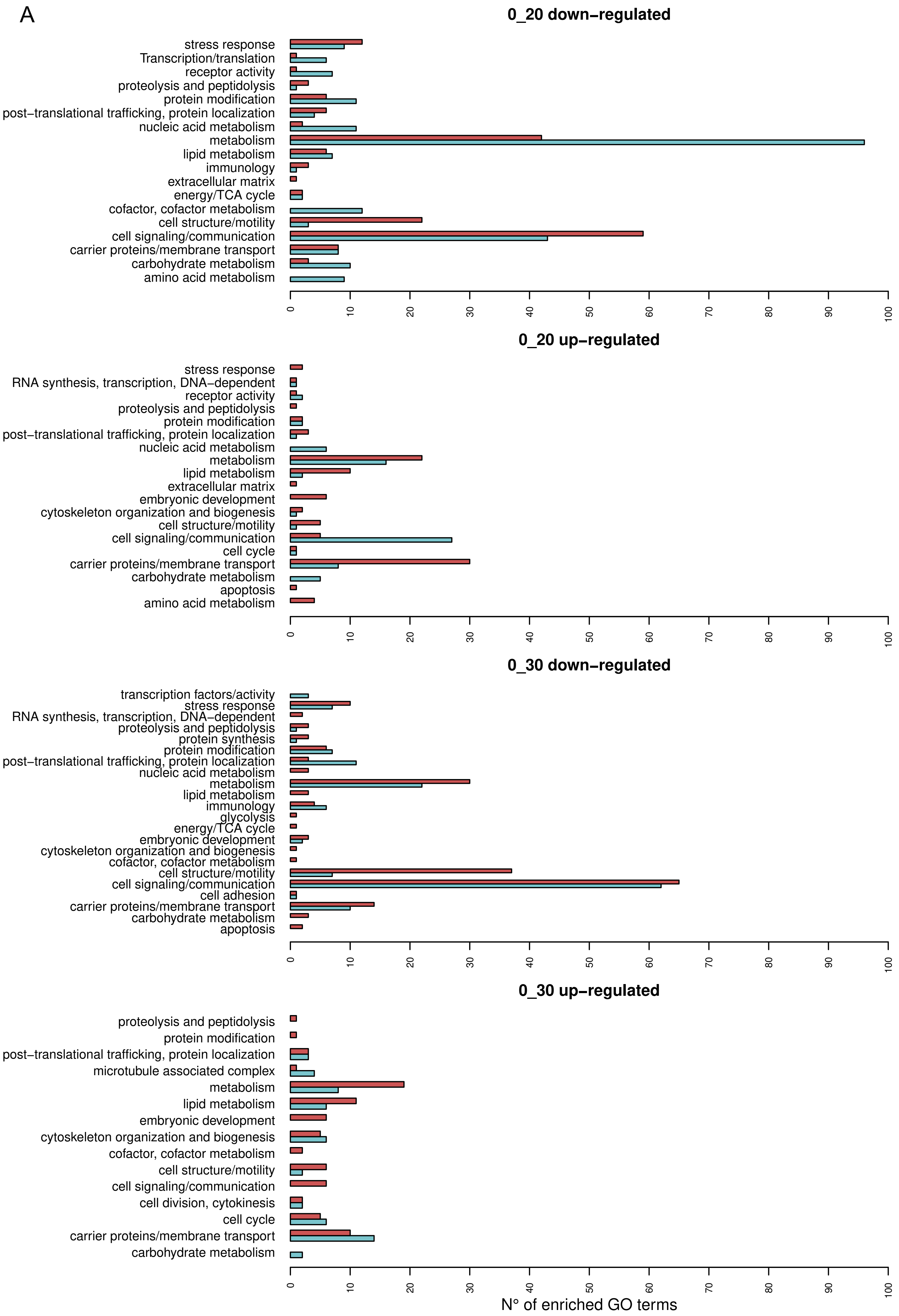

A
0_20 down−regulated
stress response
Transcription/translation
receptor activity
proteolysis and peptidolysis
protein modification
post−translational trafficking, protein localization
nucleic acid metabolism
metabolism
lipid metabolism
immunology
extracellular matrix
energy/TCA cycle
cofactor, cofactor metabolism
cell structure/motility
cell signaling/communication
carrier proteins/membrane transport
carbohydrate metabolism
amino acid metabolism
0_20 up−regulated
stress response
RNA synthesis, transcription, DNA−dependent
receptor activity
proteolysis and peptidolysis
protein modification
post−translational trafficking, protein localization
nucleic acid metabolism
metabolism
lipid metabolism
extracellular matrix
embryonic development
cytoskeleton organization and biogenesis
cell structure/motility
cell signaling/communication
cell cycle
carrier proteins/membrane transport
carbohydrate metabolism
apoptosis
amino acid metabolism
0_30 down−regulated
transcription factors/activity
stress response
RNA synthesis, transcription, DNA−dependent
proteolysis and peptidolysis
protein synthesis
protein modification
post−translational trafficking, protein localization
nucleic acid metabolism
metabolism
lipid metabolism
immunology
glycolysis
energy/TCA cycle
embryonic development
cytoskeleton organization and biogenesis
cofactor, cofactor metabolism
cell structure/motility
cell signaling/communication
cell adhesion
carrier proteins/membrane transport
carbohydrate metabolism
apoptosis
0_30 up−regulated
proteolysis and peptidolysis
protein modification
post−translational trafficking, protein localization
microtubule associated complex
metabolism
lipid metabolism
embryonic development
cytoskeleton organization and biogenesis
cofactor, cofactor metabolism
cell structure/motility
cell signaling/communication
cell division, cytokinesis
cell cycle
carrier proteins/membrane transport
carbohydrate metabolism
0 10 20 30 40 50 60 70 80 90 100
N° of enriched GO terms

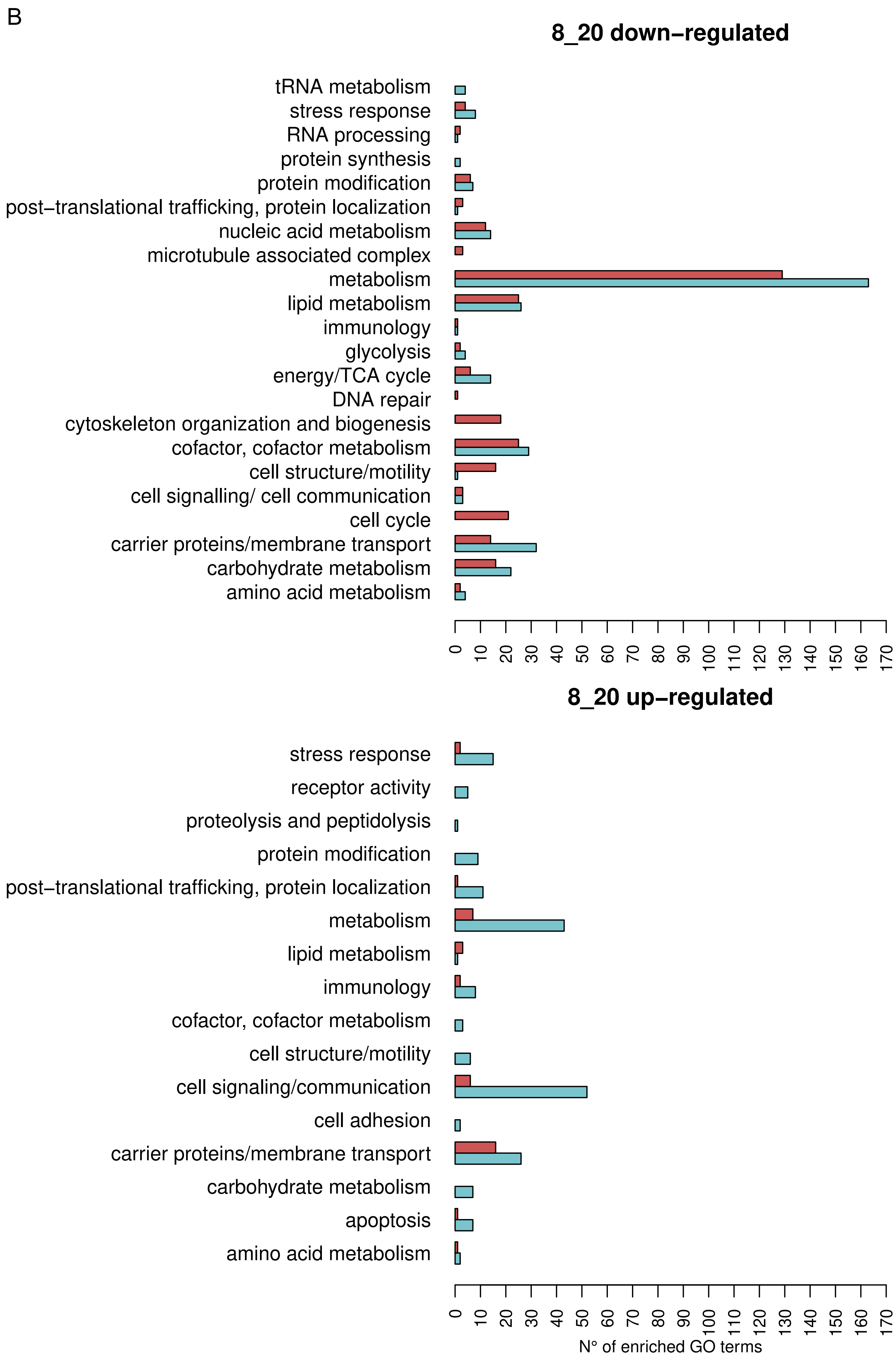
B
8_20 down–regulated
tRNA metabolism
stress response
RNA processing
protein synthesis
protein modification
post–translational trafficking, protein localization
nucleic acid metabolism
microtubule associated complex
metabolism
lipid metabolism
immunology
glycolysis
energy/TCA cycle
DNA repair
cytoskeleton organization and biogenesis
cofactor, cofactor metabolism
cell structure/motility
cell signalling/ cell communication
cell cycle
carrier proteins/membrane transport
carbohydrate metabolism
amino acid metabolism
0 10 20 30 40 50 60 70 80 90 100 110 120 130 140 150 160 170
8_20 up–regulated
stress response
receptor activity
proteolysis and peptidolysis
protein modification
post–translational trafficking, protein localization
metabolism
lipid metabolism
immunology
cofactor, cofactor metabolism
cell structure/motility
cell signaling/communication
cell adhesion
carrier proteins/membrane transport
carbohydrate metabolism
apoptosis
amino acid metabolism
0 10 20 30 40 50 60 70 80 90 100 110 120 130 140 150 160 170
N° of enriched GO terms

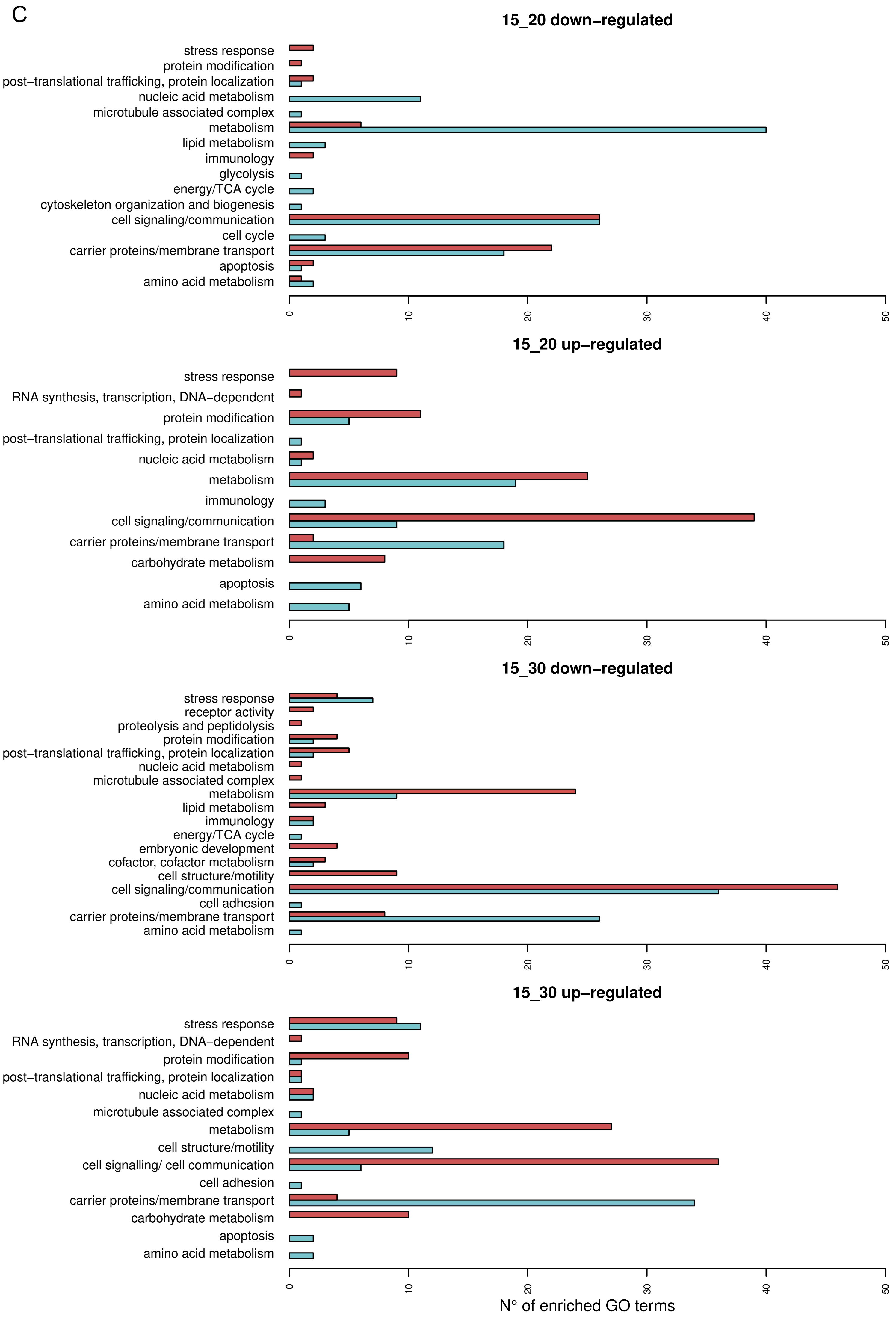
C
15_20 down−regulated
stress response
protein modification
post−translational trafficking, protein localization
nucleic acid metabolism
microtubule associated complex
metabolism
lipid metabolism
immunology
glycolysis
energy/TCA cycle
cytoskeleton organization and biogenesis
cell signaling/communication
cell cycle
carrier proteins/membrane transport
apoptosis
amino acid metabolism
0
10
20
30
40
50
15_20 up−regulated
stress response
RNA synthesis, transcription, DNA−dependent
protein modification
post−translational trafficking, protein localization
nucleic acid metabolism
metabolism
immunology
cell signaling/communication
carrier proteins/membrane transport
carbohydrate metabolism
apoptosis
amino acid metabolism
0
10
20
30
40
50
15_30 down−regulated
stress response
receptor activity
proteolysis and peptidolysis
protein modification
post−translational trafficking, protein localization
nucleic acid metabolism
microtubule associated complex
metabolism
lipid metabolism
immunology
energy/TCA cycle
embryonic development
cofactor, cofactor metabolism
cell structure/motility
cell signaling/communication
cell adhesion
carrier proteins/membrane transport
amino acid metabolism
0
10
20
30
40
50
15_30 up−regulated
stress response
RNA synthesis, transcription, DNA−dependent
protein modification
post−translational trafficking, protein localization
nucleic acid metabolism
microtubule associated complex
metabolism
cell structure/motility
cell signalling/ cell communication
cell adhesion
carrier proteins/membrane transport
carbohydrate metabolism
apoptosis
amino acid metabolism
0
10
20
30
40
50
N° of enriched GO terms
