# Supplementary material for: Is geographical variation driving the transcriptomic responses to multiple stressors in the kelp Saccharina latissima?
Source: BMC Plant Biol. 2019 Nov 21;19:513. doi: 10.1186/s12870-019-2124-0 (PMC6881991; doi:10.1186/s12870-019-2124-0)
Supplement: Supplementary file 7 — Additional file 7. Manual classification of DEGs compared to the control (8_30) according to functional categories of interest. ↑ indicates up-regulated DEGs, ↓ down-regulated DEGs. [file 12870_2019_2124_MOESM7_ESM.pdf]

Additional file 7: Manual classification of DEGs compared to the control (8_30) according to functional categories of interest. ↑ indicates up-regulated DEGs, ↓ down-regulated DEGs

		**Roscoff**					**Spitsbergen**				
		0_20	0_30	8_20	15_20	15_30	0_20	0_30	8_20	15_20	15_30
Photosynthetic components	↑	2					1	1			1
	↓	13	1	28	3	2	5	1	18		2
Photosynthesis	↑	2		2	1						
	↓	12		27	3		3	2	25		
Stress response	↑	9		13	8	2	8	8	7		
	↓	12	1	9	6	3	2		10	11	16
Transport/ cell wall synthesis, reorganization	↑	16	2	8	8	3	8	1	9	1	
	↓	29		14	10	5	14	3	25	10	15
Lipid metabolism	↑	3	1	1	1	1	3	2		1	
	↓	12		4	3	2	3		9	1	
Nucleic acid	↑	7		1	2	1	2		3	1	

metabolism	↓	5		6	4	1	1		8		
Protein degradation	↑	3		1	2	1					
	↓	7		2	1	1	5		3		
Signaling	↑	7		5			3	3	2	2	3
	↓	28	3	4	3	3	7	3	12	3	9
Transcription/Translation	↑	11			3		2	1	3		
	↓	29		19	2		3	2	19		1
Carbohydrate metabolism	↑										
	↓	4		4	4	1			4		
Aminoacid metabolism	↑			1							
	↓	6		4	2	1			1	2	1
