# Supplementary figures and images for: Is geographical variation driving the transcriptomic responses to multiple stressors in the kelp Saccharina latissima?
Source: BMC Plant Biol. 2019 Nov 21;19:513. doi: 10.1186/s12870-019-2124-0 (PMC6881991; doi:10.1186/s12870-019-2124-0)

## Slide 1
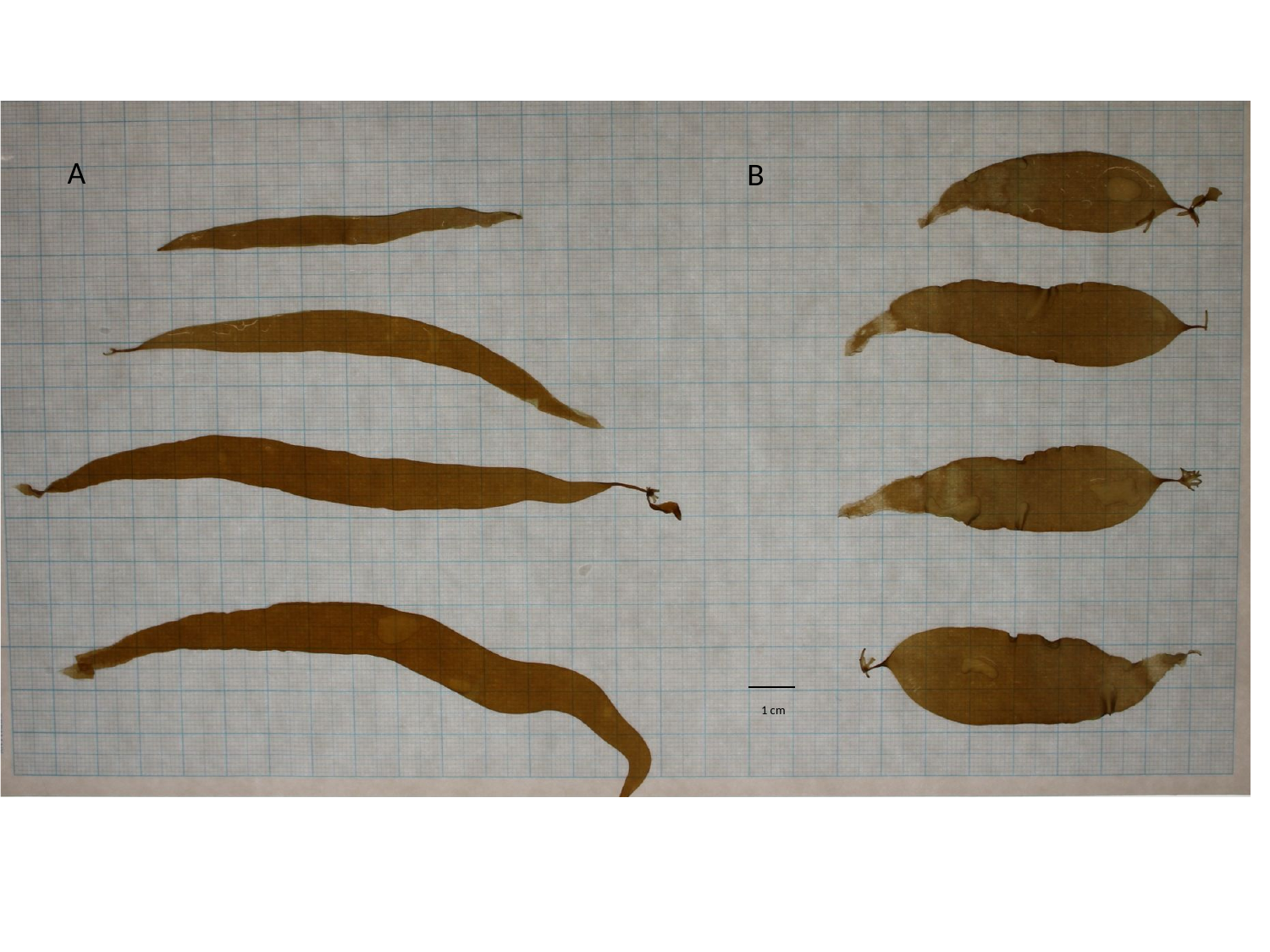

A
B
1 cm

Supplement: Supplementary file 10 — Additional file 10. Phenotypic differences between sporophytes from Roscoff and Spitsbergen before the start of the experiment. A) Spitsbergen, B) Roscoff. [file 12870_2019_2124_MOESM10_ESM.pptx]
